# Effectiveness of Long-Term Shichimotsukokato Administration on the eGFR Slope in Patients With Chronic Kidney Disease: A Retrospective Single-Arm Study

**DOI:** 10.7759/cureus.98560

**Published:** 2025-12-06

**Authors:** Kazushi Uneda, Yoshihiro Sato, Naoyuki Furukawa, Akira Kaneko, Tadamichi Mitsuma, Shigeatsu Hashimoto, Eiichi Tahara

**Affiliations:** 1 Department of Kampo Medicine, Aizu Medical Center, Fukushima Medical University, Aizuwakamatsu, JPN; 2 Department of Internal Medicine, Tenjinbashi Clinic, Fukushima, JPN; 3 Department of Endocrinology, Metabolism, Diabetology and Nephrology, Aizu Medical Center, Fukushima Medical University, Aizuwakamatsu, JPN

**Keywords:** chronic kidney disease (ckd), complementary and integrative medicine, japanese traditional (kampo) medicine, kampo formula, shichimotsukokato

## Abstract

Introduction

The prevalence of chronic kidney disease (CKD) continues to rise worldwide. Patients with CKD are exposed to increased risks of end-stage kidney disease, cardiovascular events, and mortality. Despite recent pharmacological advances, therapeutic options for CKD remain limited. Shichimotsukokato (SCMKT), a representative Kampo formula in Japanese Traditional Medicine composed of seven crude drugs, has historically been used to treat CKD. Experimental studies have demonstrated its renoprotective and antihypertensive effects. However, the clinical evidence of SCMKT for CKD patients remains limited. To evaluate the effectiveness of the long-term SCMKT administration on renal function, proteinuria, and blood pressure in patients with CKD, we conducted a retrospective, single-arm, observational study.

Methods

This study targeted outpatients with CKD who were treated with SCMKT. The study was conducted at Fukushima Medical University Aizu Medical Center and Tenjinbashi Clinic from April 2019 to May 2025 (UMIN000058358). Eligible patients were aged ≥18 years, had been prescribed SCMKT continuously for one year, and had available estimated glomerular filtration rate (eGFR) data from one year before initiation, at initiation, and one year after the initiation of SCMKT. The primary endpoint was the change in the eGFR slope between the pre-treatment and post-treatment periods. The pre-treatment eGFR slope was determined from one year before the initiation of SCMKT, and the post-treatment slope from initiation to one year after. The eGFR slope was calculated using the least-squares regression method. Secondary endpoints included changes in systolic and diastolic blood pressure, qualitative proteinuria, and the occurrence of adverse events.

Results

A total of 21 patients who were prescribed SCMKT were screened from both institutions. Finally, 12 patients were included in our study (mean age, 77.8 years; male, seven patients (58.3%)). The mean baseline eGFR was 38.7 ± 10.0 mL/min/1.73 m², and 9 patients (75.0 %) were classified as CKD stage G3b. Hypertensive nephrosclerosis was the most common primary cause of CKD. There were 11 patients (91.7 %) with concomitant hypertension, and 9 patients (75.0 %) were prescribed renin-angiotensin-aldosterone system (RAAS) inhibitors. The mean daily dose of SCMKT (Tsumura & Co., Tokyo, Japan) was 5.6 g. The mean eGFR slope significantly improved after SCMKT administration compared with the pre-treatment period (pre-treatment vs. post-treatment, −4.9 ± 7.3 vs. 5.4 ± 5.4 mL/min/1.73 m²/year; *P* = 0.002). In contrast, there were no significant changes in systolic or diastolic blood pressure (132.9 ± 15.5 vs. 135.2 ± 13.4 mmHg; 70.2 ± 13.2 vs. 73.1 ± 7.5 mmHg, respectively), and qualitative urine protein did not show a marked change. No clinical information on serious adverse events that were potentially associated with SCMKT was available. Sensitivity analyses excluding patients with changes in RAAS inhibitor therapy confirmed the robustness of our findings.

Conclusions

The one-year prescription of SCMKT was safe and may help preserve the eGFR slope in patients with CKD. Further prospective studies are warranted to confirm the renoprotective effects of SCMKT.

## Introduction

The prevalence of chronic kidney disease (CKD) continues to rise worldwide. Patients with CKD are exposed to increased risks of end-stage kidney disease, cardiovascular events, and mortality [1-3]. Recent drug developments, including sodium-glucose cotransporter-2 inhibitors or mineralocorticoid receptor antagonists, have demonstrated therapeutic efficacy in improving the CKD prognosis [4,5]. However, treatment strategies for CKD patients remain limited. Recently, the estimated glomerular filtration rate (eGFR) slope has been recognized as an important prognostic indicator for CKD [6,7], serving as a validated surrogate endpoint for CKD in clinical trials [8].

Japanese Traditional Medicine (JTM) has historically been used to treat CKD [9]. In Japan, 148 ethical Kampo formulas, which are traditional medications in JTM, are covered by the national health insurance system. Therefore, 86.7% of Japanese physicians prescribe Kampo formulas in their clinical practice [10]. Shichimotsukokato (SCMKT), a Kampo formula developed in Japan (http://mpdb.nibiohn.go.jp/stork/), has been prescribed for patients with kidney diseases and hypertension. Previous experimental studies have demonstrated the renoprotective and antihypertensive effects of SCMKT [11,12]. However, no clinical studies have analyzed the effect of the long-term administration of SCMKT on renal function and blood pressure. Therefore, we performed a retrospective study to evaluate the longitudinal effect of SCMKT on the eGFR slope, proteinuria, and blood pressure in CKD patients.

## Materials and methods

Study design

**Figure 1:**
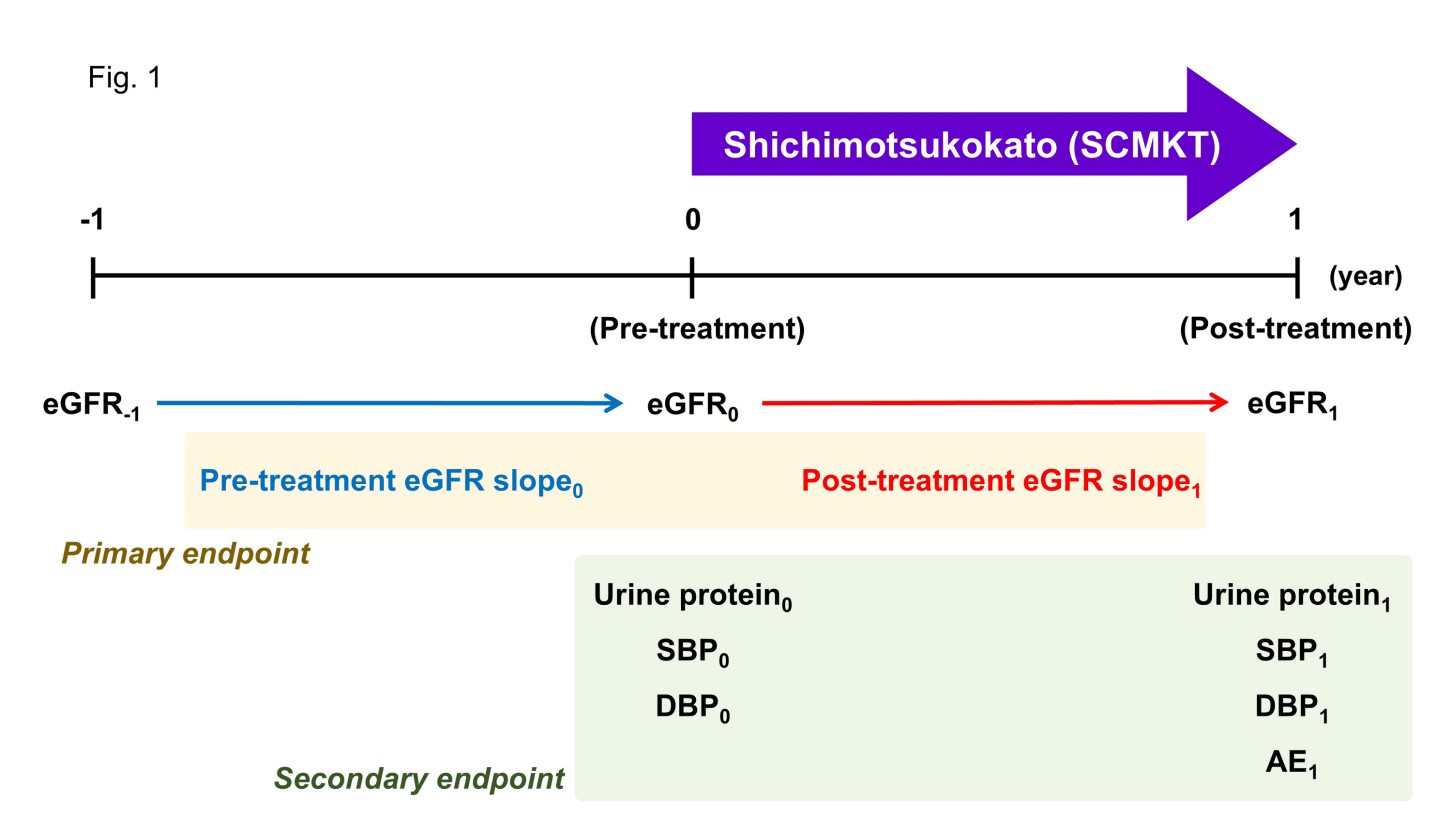
Flow chart of the study eGFR, glomerular filtration rate; SBP, systolic blood pressure; DBP, diastolic blood pressure; AE, adverse events.

To investigate the efficacy of SCMKT on renal function, proteinuria, and blood pressure in CKD patients, we conducted a retrospective, single-arm, observational study (Figure 1). The study period was from April 2019 to May 2025. The study protocol was approved by Fukushima Medical University Ethics Committee (REC2025-013) and registered at the UMIN Clinical Trials Registry (UMIN000058358).

Participants

We targeted outpatients at Fukushima Medical University Aizu Medical Center and Tenjinbashi Clinic. The inclusion criteria were 1. age≥18 years old; 2. diagnosis of CKD according to the criteria of the Japan Society of Nephrology [13]; 3. continuous administration of SCMKT for one year; 4. availability of eGFR data at the initiation of SCMKT, one year prior to its initiation, and one year after its initiation. CKD was diagnosed when either (i) or (ii) or both persist for over three months: (i) Evidence of kidney injury on abnormal urinalysis (especially proteinuria ≥0.15 g/gCr (albuminuria ≥30 mg/gCr)), imaging, blood tests, or pathological information. (ii) GFR <60 mL/min/1.73 m². eGFR values were calculated from serum creatinine using the Japanese GFR estimation formula [13]. Patients who did not provide consent to this study were excluded.

In addition, hypertension was defined as an office blood pressure ≥140/90 mmHg or a home blood pressure ≥135/85 mmHg, in accordance with the Guidelines for the Management of Elevated Blood Pressure and Hypertension 2025 [14].

Endpoints

The primary outcome was the changes in the eGFR slope before and after SCMKT initiation in the targeted CKD patients. eGFR slopes were calculated using ordinary least-squares linear regression models [15]. For each patient, all available eGFR values during the observation period were aligned with their corresponding time points, measured relative to the baseline assessment. A linear regression was performed for each patient, and the estimated annual change in eGFR from that model was used as the individual eGFR slope value. The pre-treatment eGFR slope was determined using all eGFR data from one year before SCMKT administration up to the day of administration. The post-treatment eGFR slope was calculated using all eGFR data from the administration day to one year after.

The secondary outcomes included (i) the changes in systolic and diastolic blood pressure before and after SCMKT initiation, (ii) the qualitative changes in proteinuria before and after SCMKT initiation, and (iii) the adverse events potentially related to SCMKT initiation.

Statistical analysis

Descriptive statistics were presented as means with standard deviations for continuous variables and frequencies with percentages for categorical variables. Continuous values were analyzed by the paired *t* analysis. For qualitative urine tests, data were analyzed by the Wilcoxon signed-rank test. *P* values <0.05 indicated statistical significance. Statistical analyses were performed using SPSS, version 29.0 (IBM, Armonk, NY, USA).

## Results

Patient characteristics

A total of 21 patients who were prescribed SCMKT were screened at both institutions. Finally, 12 patients were included in our study. Table 1 shows the patients' characteristics. The average age was 77.8 years old. There were 7 (58.3%) male patients. The average value of eGFR at the administration of SCMKT was 38.7±10.0 mL/min/1.73m^2^. There were nine patients (75.0%) categorized as CKD stage G3b. Hypertensive nephrosclerosis was the most common primary disease in our study, and 11 patients (91.7%) had concomitant hypertension. Renin-angiotensin-aldosterone system (RAAS) inhibitors were prescribed in 9 patients (75.0 %). In our study, all SCMKT prescriptions were manufactured by Tsumura & Co. (Tokyo, Japan), and the average dose of SCMKT was 5.6 g/day.

**Table 1:** Baseline characteristics at the initiation of shichimotsukokato treatment (n=12) Values are presented as mean ± standard deviation or number (%). CKD, chronic kidney disease; eGFR, estimated glomerular filtration rate; RAAS, renin-angiotensin-aldosterone system; SGLT-2, sodium-glucose cotransporter-2

Age	77.8±9.0
Male, n (%)	7 (58.3)
Body mass index, kg/m^2^	24.9±5.5
CKD stage, n (%)	
G3a	2 (16.7)
G3b	9 (75.0)
G4	1 (8.3)
Primary disease of CKD, n (%)	
Hypertensive nephrosclerosis	7 (58.3)
Diabetic kidney disease	2 (16.7)
Lupus nephritis	1 (8.3)
Idiopathic membranous nephropathy	1 (8.3)
Unilateral nephrectomy	1 (8.3)
Comorbidities, n (%)	
Hypertension	11 (91.7)
Diabetes mellitus	3 (25.0)
Dyslipidemia	6 (50.0)
Cardiovascular disease	3 (25.0)
Systolic blood pressure, mmHg	132.9±15.5
Diastolic blood pressure, mmHg	70.2±13.2
eGFR, mL/min/1.73m^2^	38.7±10.0
Concomitant medications, n (%)	
RAAS inhibitors	9 (75.0)
Diuretics	2 (16.7)
SGLT-2 inhibitors	0 (0)
Statins	6 (50.0)
Number of antihypertensive drugs	2.5±1.8

The patients' laboratory and urinary data are shown in Table 2. The average values of low-density lipoprotein cholesterol and hemoglobin A1c were 108.3 mg/dL and 5.9 %, respectively. Although urinary data were missing in three cases, qualitative urine protein was negative in only two patients.

**Table 2:** Blood and urine test results at the initiation of shichimotsukokato treatment (n=12) Values are presented as mean ± standard deviation or number (%). Urine test data were available for nine patients. eGFR, estimated glomerular filtration rate; HDL-C, high-density lipoprotein cholesterol; LDL-C, low-density lipoprotein cholesterol

Blood and urine tests	Results	Reference range
Hemoglobin, g/dL	13.3±1.1	11.6-14.8
Serum creatinine, mg/dL	1.3±0.4	0.46-0.79
eGFR, mL/min/1.73m^2^	38.7±10.0	≥60
Uric acid, mg/dL	6.1±10.0	2.6-6.9
Triglycerides, mg/dL	130.8±60.9	30-149
HDL-C, mg/dL	56.1±17.4	40.0-103.0
LDL-C, mg/dL	108.3±19.4	65-139
Hemoglobin A1c, %	5.9±0.6	4.7-6.1
Urine protein		Negative
-	2 (22.2)	
±	1 (11.1)	
+	3 (33.3)	
≥2+	3 (33.3)	
Urine occult blood	7 (77.8)	Negative
-	1 (11.1)	
±	1 (11.1)	
+		
Urine glucose		Negative
-	8 (88.9)	
3+	1 (11.1)	

Improvement in the eGFR slope after SCMKT administration

Table 3 shows the changes in the eGFR slope and qualitative urinary protein in patients before and after SCMKT administration. The eGFR slope significantly improved after the SCMKT treatment compared with the pre-treatment period (pre-treatment vs. post-treatment -4.9±7.3 vs. 5.4±5.4, *P*=0.002). On the other hand, there was no observable difference in the qualitative urine protein before and after SCMKT treatment (*P*=0.157).

**Table 3:** Changes in the eGFR slope, urine protein, and blood pressure before and after shichimotsukokato treatment (n=12) Values are presented as mean ± standard deviation or number (%). Urine protein data were available for nine patients. eGFR, estimated glomerular filtration rate

Parameter	Pre-treatment	Post-treatment	*P* value
eGFR slope, mL/min/1.73m^2^/year	-4.9±7.3	5.4±5.4	0.002
Systolic blood pressure, mmHg	132.9±15.5	135.2±13.4	0.445
Diastolic blood pressure, mmHg	70.2±13.2	73.1±7.5	0.461
Urine protein			0.157
-	1	1	
±	1	0	
+	3	3	
≥2+	1	2	

Blood pressure evaluation

Table 3 also shows the changes in blood pressure before and after one year of treatment with SCMKT. There was no significance in blood pressure before and after SCMKT administration (systolic blood pressure, 132.9±15.5 vs. 135.2±13.4, *P*=0.445; diastolic blood pressure, 70.2±13.2 vs. 73.1±7.5, *P*=0.461).

Adverse events

During the study period, we were not able to extract the clinical information on serious adverse events that may be associated with SCMKT.

Sensitivity analysis

During the study period, the types or doses of RAAS inhibitors were changed in four patients. To confirm the robustness of our results, we excluded them and conducted additional analysis (Table 4). The change in the eGFR slope remained statistically significant, whereas systolic and diastolic blood pressures showed no significant differences during SCMKT treatment (eGFR slope, -4.5±7.4 vs. 5.1±5.3, *P*=0.026; systolic blood pressure, 131.8±18.3 vs. 132.4±14.9, P=0.880; diastolic blood pressure, 72.3±15.4 vs. 76.5±5.6, *P*=0.481). In addition, qualitative urine protein did not show a statistical change (*P*=0.157).

**Table 4:** Sensitivity analysis excluding patients with changes in RAAS inhibitor therapy (n=8) Values are presented as mean ± standard deviation or number (%). Urine protein data were available for five patients. RAAS, renin-angiotensin-aldosterone system; eGFR, estimated glomerular filtration rate

Parameter	Pre-treatment	Post-treatment	*P* value
eGFR slope, mL/min/1.73m^2^/year	-4.5±7.4	5.1±5.3	0.026
Systolic blood pressure, mmHg	131.8±18.3	132.4±14.9	0.88
Diastolic blood pressure, mmHg	72.3±15.4	76.5±5.6	0.481
Urine protein			0.157
-	1	1	
±	1	0	
+	3	3	
≥2+	1	2	

## Discussion

This is the first study to report the long-term effectiveness of SCMKT in preserving the eGFR slope in patients with CKD. A previous case report documented that a three-month course of SCMKT therapy improved renal function in a patient with CKD [16]. In this study, one-year treatment with SCMKT resulted in a significant improvement in the eGFR slope among patients with CKD.

SCMKT, a Kampo formula developed by the Japanese physician Keisetsu Otsuka, is composed of seven crude drugs, including Astragalus root (*Astragali radix*), Uncaria hook (*Uncariae Ramulus Cum Uncis*), Peony root (*Paeoniae Radix*), Japanese Angelica root (*Angelicae Acutilobae Radix*), Rehmannia root (*Rehmanniae Radix*), Cnidium rhizome (*Cnidii Rhizoma*), and Phellodendron bark (*Phellodendri Cortex*) (http://mpdb.nibiohn.go.jp/stork/). Among these crude drugs, Astragalus root and Uncaria hook are considered key components for the pathophysiology of CKD [17,18]. The high-performance liquid chromatography profile of SCMKT showed the presence of characteristic chemicals, including astragaloside IV, hirsutine, and isorhynchophylline [11,19]. Previous experimental studies have shown that these phytochemicals inhibit podocyte injury, mitochondrial damage, and interstitial fibrosis in CKD models [20-24].

Furthermore, several clinical studies have demonstrated that the prescription of Astragalus root or Kampo formulas containing it preserves the serum creatinine levels or eGFR in CKD patients during treatment periods ranging from six months to four years [25,26]. A recent propensity-matched cohort study at a single center in Japan also reported that long-term administration of various Kampo formulas preserved the eGFR slope in CKD patients [27]. Although SCMKT was infrequently prescribed in that study, 75.0% of participants received Kampo formulas containing Astragalus root. These findings are consistent with our results and suggest a potential protective effect of long-term Kampo formula administration on CKD progression.

On the other hand, we did not observe any improvement in proteinuria, although we recently reported a case showing the effectiveness of SCMKT in an older patient with diabetic nephrotic syndrome [28]. Some studies have reported that Astragalus root exerts pharmacological benefits in reducing proteinuria in CKD and experimental models [20,29]. The discrepancy may be attributed to the qualitative urinalysis in the present study. Additionally, we did not observe the changes in blood pressure among the patients. Although SCMKT has been reported to reduce blood pressure in hypertensive rat models [11,12], patients in our study had already been prescribed multiple antihypertensive drugs, which may have influenced our outcomes. Further investigations are warranted to clarify the effectiveness of SCMKT on proteinuria and hypertension.

This study has several limitations. First, we could not exclude several biases, including selection and information biases, because our study was a retrospective single-arm cohort design. Second, regression analysis could not be performed owing to the small sample size. Third, the interaction between SCMKT and other Western medications remains unclear. Fourth, we were unable to estimate the predictive intervals of the eGFR slope because the number of eGFR measurements was insufficient in some cases.

## Conclusions

The one-year prescription of SCMKT was safe and may help preserve the eGFR slope in patients with CKD. Further prospective studies are warranted to confirm the renoprotective effects of SCMKT.
